# Pacemaker Implantation in Juvenile Neuronal Ceroid Lipofuscinosis (CLN3)–A Long-Term Follow-Up Study

**DOI:** 10.3389/fneur.2022.846240

**Published:** 2022-03-10

**Authors:** Mette Møller Handrup, Henning Mølgaard, Brian N. Andersen, John R. Ostergaard

**Affiliations:** ^1^Department of Pediatrics and Adolescent Medicine, Centre for Rare Diseases, Aarhus University Hospital, Aarhus, Denmark; ^2^Department of Cardiology, Aarhus University Hospital, Aarhus, Denmark

**Keywords:** Juvenile Neuronal Ceroid Lipofuscinosis, CLN3, neurodegenerative diseases, sick sinus syndrome, pacemaker

## Abstract

It is well documented that deteriorating heart function due to deposition of ceroid lipopigment is a significant co-morbidity in Juvenile Neuronal Ceroid Lipofuscinosis (CLN3 disease) although the exact disease mechanisms remain unknown in any NCL form. An increasing frequency of cardiac conduction disorders including severe bradycardia and sinus arrest is seen in the late teens, as is a left ventricular hypertrophy in the early 20s. Only a few case reports of pacemaker implantation have been published, and so far, no long-term follow-up study exists. As new treatment options emerge, more patients will live longer and the need for pacemaker will likely increase, why knowledge of long-term outcome is needed. In the present study, we present the course of six patients from the original Danish CLN3-heart population study (*n* = 29) published in 2011 in whom pacemaker implantation was indicated from a cardiac point of view. In two cases, the families deselected pacemaker implantation. In four males, aged 19-29 years, all having a good general condition, a dual-chamber pacemaker (St. Jude Medical™ Accent/Assurity MRI™) was implanted in general anesthesia without any complications. At follow-up 9 years later, three were still alive. According to the parents' opinion they still have a good quality of life, now 26, 30, and 36 years old. Pacemaker treatment is safe and may have great impact on quality of life. However, the medical indication for pacemaker treatment is relative and it is important that various aspects, including the patient's general condition and family preferences, are thoroughly discussed before making the final decision.

## Introduction

The Neuronal Ceroid Lipofuscinoses (NCL) is a group of hereditary diseases characterized by dysfunction of the lysosomes. Over time, increasing deposition of ceroid lipopigment leads to increasing degeneration and loss of function of the cells in question. There are today 14 different subtypes that all but one debut in childhood.

The Juvenile Neuronal Ceroid Lipofuscinosis, also called CLN3 disease, is a fatal neurodegenerative disease with an estimated incidence range from 0.2 to 7.0 per 100,000 (1). It is an autosomal recessive disorder caused by a defect in the *CLN3* gene, which in the majority of cases (>85%) is due to a homozygous deletion of exon 7/8 (2). The disease initially presents with visual impairments and within a few years, decline of cognitive and motor function, behavioral changes and epilepsy follow. As the disease initially presents with degenerative changes of the nervous system, therapeutics and research have mainly been concentrated concerning pathology changes of the CNS, but in recent years it has been well documented, that other organ systems, such as the heart, are also affected (3–6). Accumulation of storage material has been reported in CLN3 disease, but knowledge of the exact disease mechanisms in any NCL form is still lacking (3). In 2011, a Danish clinical cross-sectional and follow-up study was published comprising 29 patients with CLN3 (7). In this study, progressive cardiac impairment including repolarization disturbances, ventricular hypertrophy and sinus node dysfunction leading to severe bradycardia and other conduction abnormalities were reported. Inverted T waves were present from 14 years of age and were associated with an increased risk of early death. Bradycardia and left ventricular hypertrophy were found in all patients > 20 years old, and in one patient pacemaker treatment was indicated from a cardiologic point of view and in question. So far, pacemaker implantation in CLN3 disease has only been reported in case studies (4, 5), and no long-term follow-up studies exist. As new treatment options emerge, including gene therapy (8, 9), CLN3 patients may live longer and the need for pacemaker will likely increase. Thus, knowledge of long-term outcome following pacemaker implantation is needed.

In the present follow-up study of the original Danish CLN3 heart population (7), we describe indications, reflections and course of six patients having a severe reduction in heart rate, long periods of sinus arrests and gradually accompanying clinical symptoms of cardiac failure and/or sudden loss of consciousness and muscular tone different from their habitual seizures.

## Materials and Methods

### Participants

For more than 20 years, all Danish patients with CLN3 disease have been regularly treated at one national site, i.e., Centre for Rare Diseases, Aarhus University Hospital. The Centre is tax financed and monitors patients of all types of NCL from infancy until death, regardless of severity of disease and socioeconomic background. In 2003-2010, 29 of 30 patients with CLN3 in Denmark participated in a cross-sectional and follow-up study where 24 h Holter examination, a 12-lead ECG and echocardiography were performed every second year. The study was published in 2011 (7). Since then, all patients < 18 years of age having a reduced minimum heart rate (<40 beats per min) or a reduced mean heart rate (<55 beats per min), and patients above 18 years of age regardless of heart rate, have been offered a yearly 24 h Holter examination. In the present study we included those patients who were eligible for pacemaker implantation if they fulfilled the following criteria:

1) Clinical symptoms of cardiac failure (shortness of breath, severe fatigue, peripheral cyanosis and/or syncope/near-syncope) and SA node dysfunction.

2) A 24 h Holter examination demonstrated severe bradycardia and/or periods of prolonged sinus arrest, which were considered abnormal and explanatory for the above clinical symptoms.

In all patients an echocardiography was performed. In patients who fulfilled the above criteria and where caregivers after thorough information agreed to the procedure, a dual chamber pacemaker (St. Jude Medical™ Accent/Assurity MRI™) was subsequently implanted in general anesthesia.

The present study was conducted as a follow-up study based on both prospective and retrospective, descriptive data. A medical chart review was performed, and data was collected for each patient. The data included information about genetics, demographic characteristics, sex, age and clinical status at the time of assessment for 24 h Holter examination, echocardiography, and pacemaker implantation. Patients were followed until end of study period (August 2021) or death.

### Standard 12-Lead ECG Recording

Below 12 years, negative T waves in lead V2 and V3 were considered normal. Above 12 years, the standard criteria for normality were applied. Abnormal findings included complete right bundle branch block, left ventricular hypertrophy, flat or deep negative T waves in V2–V6, pathologic Q waves in lead III, grade I atrioventricular (AV) block, grade II type II AV block, and permanent atrial fibrillation or permanent atrial flutter.

### Twenty-Four-Hour Ambulatory ECG Recording (Holter Examination)

To exclude periods with artifacts, each recording was analyzed interactively by an experienced technician. Heart rate (HR) was measured as HR per 1-min period [beats per minute (bpm)]. Maximum HR (HR_max, 24h_) and minimum HR (HR_min, 24h_) were identified, and mean 24-h HR (HR_mean, 24h_) was calculated. Based on values for 95% confidence limits in healthy individuals normal values for HR_min, 24h_ and HR_mean, 24h_ were set as 40 and 55 bpm, respectively (10).

### Echocardiography

Reviewing the ECG's and Holter investigations, and making the assessment of whether or not a pacemaker implantation was indicated, was performed by the same cardiologic specialist (HM) in all the patients. Echocardiography was performed by one of three senior experienced cardiologists on a Vingmed Vivid 7 (GE Healthcare, Oslo, Norway) apparatus using a standardized protocol for evaluation of morphology. Left ventricular hypertrophy was defined as 11-mm wall thickness.

### Statistical Analyses

The study was designed as a descriptive study and no statistical analyses were performed.

## Results

**Figure 1:**
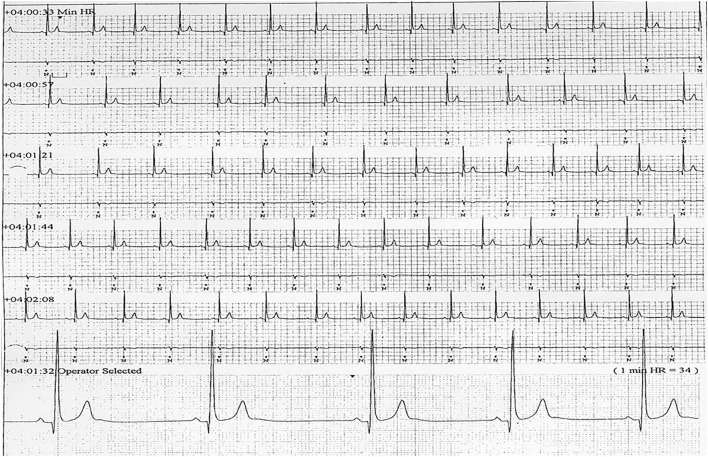
ECG transcript of patient 5, aged (26 years of age) showing sinus bradycardia (24H_min_ = 34).

**Figure 2:**
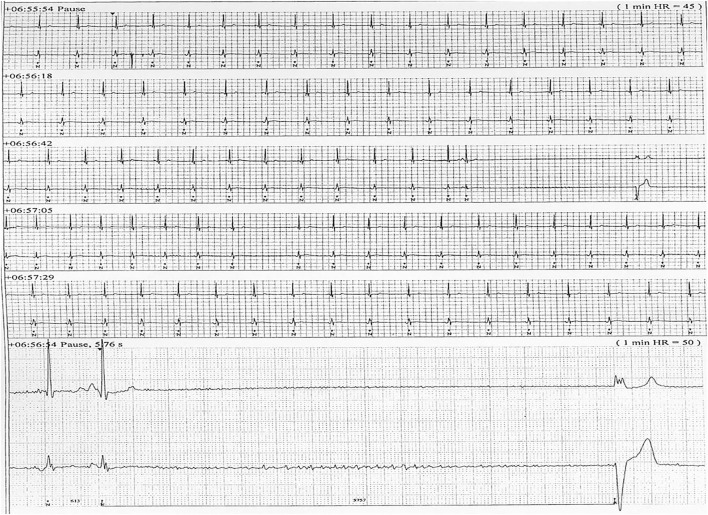
ECG transcript of patient 5, aged (26 years of age) having recurrent sinus arrests, the one shown lasting 5.76 s.

**Figure 3:**
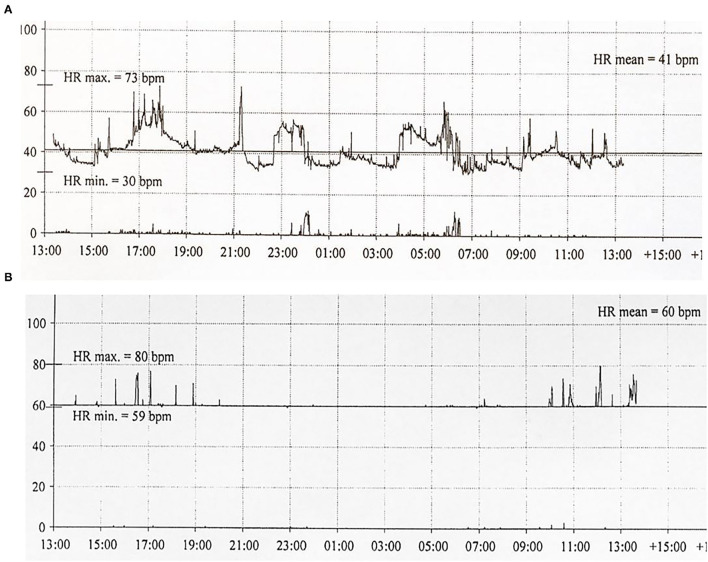
Twenty-four hour ECG transcripts of Patient 4 (21 years of age) before **(A)** and 3 months **(B)** following pacemaker implantation.

Six patients (five males) fulfilled the criteria for pacemaker implantation (Table 1). The patients were between 19 and 27 years old and all had a homozygous deletion of exon 7/8 in *CLN3*. In two cases pacemaker implantation was omitted. In one case, the procedure was refrained due to a severely affected general condition of the patient and in the second case because the parents were opposed to a possible life-prolonging treatment. All patients had clinical symptoms at the time of the pacemaker implantation. Two patients, who were still physical active, complained shortness of breath and feeling extremely tired. In the other patients, syncope and near-syncope was the primary symptom. In all patients severe sinus bradycardia (Figure 1) was demonstrated (HR_min, 24h_ between 20 and 32 beats per min), as were prolonged sinus arrests (Figure 2). Echocardiography was initially performed in four out of six patients. In two patients, aged 19 and 21 years, a normal examination was found. In the two other patients, aged 23 and 27 years, ventricular hypertrophy was demonstrated. Figure 3 shows 24 h heart rate profiles before (A) and after (B) pacemaker implantation in one of the treated patients. HR_min, 24h_ has increases from 30 to 59 bpm and HR_mean, 24h_ from 41 to 60 bpm.

**Table 1:** Clinical status at time of implantation and follow-up.

**Clinical status at pacemaker inclusion**								
**Patient number (Sex)**	**Genotype**	**Age at pacemaker implantation**	**HR mean (24 h)**	**HR min**	**ECG findings**	**Echocardiography**	**Cardiac-related symptoms**	**Clinical function**
1 (Male)	*CLN3* del (exon 7/8)	20	36	28	Sinus arrhythmia, Sinus arrest	Normal	Syncope Cold hands and feet Peripheral cyanosis	Walk independently Talk in sentences
2 (Male)	*CLN3* del (exon 7/8)	19	54	32	AV block, type 2, Sinus-arrest	Normal	Dyspnea during activity	Walk independently Talk in sentences
3 (Male)	*CLN3* del (exon 7/8)	27	38	28	Sinus-arrest	Ventricular hypertrophy	Cold hands and feet Peripheral cyanosis	Use of wheelchair Use single words No sentences
4 (Male)	*CLN3* del (exon 7/8)	21	45	30	Sinus arrest, 11 s	Not available	Tired Dyspnea	Walk independently Talk in sentences
5 (Male)	*CLN3* del (exon 7/8)	Pacemaker deselected by parents 23 years of age	37	24	Sinus arrest, 21 s	Ventricular hypertrophy	Many syncope's Very tired Peripheral cyanosis	Use of wheelchair Use single words
6 (Female)	*CLN3* del (exon 7/8)	Pacemaker deselected due to severe general condition 22 years of age	53	20	Sinus arrest, 26 s	Normal	Many syncope's Very tired Cold hands and feet Peripheral cyanosis	Use of wheelchair No words, only sounds
**Clinical status at follow-up**								
**Patient number (Sex)**	**Genotype**	**Age at follow-up**	**Follow-up (years)**	**Alive**	**Course of death**	**Echocardiography**	**Cardiac-related symptoms**	**Clinical function**
1 (Male)	*CLN3* del (exon 7/8)	30	9	Yes	NA	Ventricular hypertrophy	None	Use of wheelchair No words, only sounds
2 (Male)	*CLN3* del (exon 7/8)	26	6	Yes	NA	Ventricular hypertrophy	None	Use of wheelchair Use single words
3 (Male)	*CLN3* del (exon 7/8)	36	8	Yes	NA	Ventricular hypertrophy	None	Use of wheelchair No words, only sounds
4 (Male)	*CLN3* del (exon 7/8)	passed away at the age of 22	1	No	pneumonia	-	-	-
5 (Male)	*CLN3* del (exon 7/8)	passed away at the age of 25	2	No	pneumonia	-	-	-
6 (Female)	*CLN3* del (exon 7/8)	passed away at the age of 22	0.4	No	pneumonia	-	-	-

The patients were followed for 9 years or until they deceased. One patient died 1 year after the pacemaker implantation due to pneumonia. The other three patients were still alive and, respectively, 26, 30, and 36 years old at the end of the study period. The patients' caregivers have reported that complaints of shortness of breath, when still physical active disappeared after the pacemaker implantation, and there had been no episodes of syncope in any of the patients. They all got warm extremities following the implantation, and there were no pacemaker related infections or need for re-implantation. At follow-up, all surviving patients had ventricular hypertrophy demonstrated by ultrasound of the heart.

In one case the pacemaker implantation was omitted due to a severe general condition of the patient including intractable epilepsy. The general condition of the patient further deteriorated during the following months and the patient continued to have multiple episodes of sinus arrest of up to 26 s leading to multiple syncope fainting. Four months later the patient died of pneumonia. In the case where pacemaker implantation was omitted because the parents were opposed to a possible life-prolonging treatment, the patients died 2 years later due to pneumonia. In the meanwhile, the patient had recurrent syncope fainting, and repeated ECG's demonstrated multiple episodes of sinus arrest with increasing duration up to 21 s. Echocardiography demonstrated ventricular hypertrophy.

## Discussion

In CLN3, pronounced deposition of ceroid lipopigment has been shown to occur in the sinus node of the heart, its autonomic nerve supply, as well as in the atrioventricular node and in the bundle of His, and to a lesser extent inside the cardiomyocytes of CLN3 patients (3, 7). Accordingly, an increasing frequency of conduction disorders including sinus arrest is seen in these patients in their late teens, along with left ventricular hypertrophy in the early 20 s (7).

The experience of pacemaker treatment for CLN3 disease is limited and only case reports have been published. In 2014, two siblings with a specific, not previous described *CLN3* mutation both received a pacemaker due to severe bradycardia and sick sinus syndrome (4). Although they later received an implantable cardiac defibrillator, the cardiac function deteriorated to cardiac failure. In a single patient reported by Dilaveris et al. (5), pacemaker implantation resulted in a more alert patient, and previous reported difficulties with dysphagia and choking during mealtimes disappeared. In the present study, we describe the clinical course of six adult patients in which pacemaker implantation was indicated from a cardiac point of view. Four of them ended up having a pacemaker. Three patients were still alive 9 years later, and in all patients the caregivers reported that they have a better quality of life. Their activity levels were increased, their temperature of the peripheral extremities remained normal, and there had been no shortness of breath and no cardiac related fainting. All three patients now showed ventricular hypertrophy, and their motor and linguistic abilities continue to regress following pacemaker implantation, indicating a further ongoing progression of the underlying CLN disease.

Our follow-up cohort consists exclusively of males, which might indicate a gender difference. It might be related to the small number of participants. However, sex difference in CLN3 disease course has been reported in both a North American and Danish study (11, 12) where females demonstrated earlier loss of independent functions, had lower quality of life, and died approximately 18 months earlier. In addition, females demonstrate cardiac pathology earlier as do males (7), and as we did not recommend pacemaker implantation in patients with a severely affected general health condition, the apparent gender difference might be real.

In our study we did not have an explicit definition of what eventually should lead to pacemaker removal. One could be concerned that having a pacemaker in a progressive and ultimately lethal disease might lead to an unnecessary extended course of the disease at the terminal phase. However, one patient died due to pneumonia already 1 year following pacemaker implantation. Thus, having a pacemaker seems not to affect the real terminal phase, but we cannot exclude that pacemaker implantation is a life-prolonging procedure, and we therefore sincerely encourage to a thorough dialog with parents before decision of a potential pacemaker implantation has to be made. In two patients, both with long-lasting sinus arrests, pacemaker implantation was deselected. They both were in the terminal phase, and although it cannot be known with certainty, pacemaker treatment would hardly have extended their lives significantly.

The field of NCL research is currently evolving rapidly and progress in gene therapy may be glimpsed in the horizon (8, 9). Nevertheless, we know from the progress in CLN2 treatment that therapy may only be effective in some organ systems (13). In the close future we face gene therapy or other new treatment regimens in patients with CLN3 disease, but whether these treatments will also be effective outside the central nervous system such as the heart, still remain to be elucidated.

### Limitations

The study comprised a small number and although the patients were followed consecutively by only one or two different clinicians during follow-up, the follow-up study has a retrospective design as well. Only CLN3 patients having the common homozygous deletion of exon 7/8 in the CLN3 gene participated, and a phenotypic variability in cardiac symptoms is still to be expected. Additionally, related to our inclusion criteria for pacemaker implantation, we might have a selected a group of CLN3 patients with a relatively benign disease course.

## Conclusions

Our study confirms that severe cardiac conduction disturbances, which principally requires decision regarding pacemaker treatment, is relatively common in patients with CLN3 and leads to symptoms reducing quality of life, including syncope fainting. A pacemaker implantation is a safe procedure in CLN3 patients, and in the present case-series it improved their quality of life and prevented further episodes of syncope. We cannot exclude that the procedure may be life-prolonging and will emphasize the importance of a thorough information of parents/caregivers when decision of a potential pacemaker implantation has to be made.

## Data Availability Statement

The raw data supporting the conclusions of this article will be made available by the authors, without undue reservation.

## Ethics Statement

The studies involving human participants were reviewed and approved by the Regional Legal Office of the Central Denmark Region, Journal no 1-45-70-72-21, August 25, 2021. Written informed consent for participation was not required for this study in accordance with the national legislation and the institutional requirements.

## Author Contributions

MH and JO have conceived and designed the follow-up protocol and made first draft. JO, MH, and BA conducted the follow-up study. HM performed and evaluated the cardiac examinations. All authors have approved the final manuscript.

## Conflict of Interest

The authors declare that the research was conducted in the absence of any commercial or financial relationships that could be construed as a potential conflict of interest.

## Publisher's Note

All claims expressed in this article are solely those of the authors and do not necessarily represent those of their affiliated organizations, or those of the publisher, the editors and the reviewers. Any product that may be evaluated in this article, or claim that may be made by its manufacturer, is not guaranteed or endorsed by the publisher.
